# Assessment of trace metal alterations in the blood, cerebrospinal fluid and tissue samples of patients with malignant brain tumors

**DOI:** 10.1038/s41598-020-60774-0

**Published:** 2020-03-02

**Authors:** Aleksandar Stojsavljević, Ljiljana Vujotić, Branislav Rovčanin, Slavica Borković-Mitić, Marija Gavrović-Jankulović, Dragan Manojlović

**Affiliations:** 1 Innovation Center of the Faculty of Chemistry, University of Belgrade, Studentski trg 12-16, Belgrade, Serbia; 2 University of Belgrade – Faculty of Chemistry, Studentski trg 12-16, Belgrade, Serbia; 3 Faculty of Medicine, University of Belgrade, Clinical Center of Serbia, Belgrade, Serbia; 4 Clinical Center of Serbia, Neurosurgery Division, Dr Koste Torodorića 4, Belgrade, Serbia; 5 Center for Endocrine Surgery, Clinical Center of Serbia, Dr Subotića 13, Belgrade, Serbia; 6 Department of Physiology, Institute for Biological Research “Siniša Stanković”, University of Belgrade, Bulevar despota Stefana 142, Belgrade, Serbia

**Keywords:** Cancer, Neuroscience, Biomarkers, Oncology, Pathogenesis, Risk factors, Chemistry

## Abstract

The pathogenesis of malignant brain tumors (MBTs) should be better understood due to the evident association between prolonged exposure to metals and increased risk of MBTs. The present research aimed to find trace metals that could contribute to the pathogenesis of MBTs. Essential trace elements (Mn, Co, Zn, Cu, Se) and relevant toxic metals (Al, Ni, As, Sr, Cd, Ce, Pt, Pb, U) in the serum, cell fraction (CF), cerebrospinal fluid (CSF) and cancerous tissue (CT) samples of MBT patients were analyzed. The results were compared with sex- and age-matched control groups. For the first time, this research showed that elemental profiles of serum, CF, CSF and CT samples in MBT patients were significantly altered compared to the appropriate controls, as well as that higher contents of trace elements (particularly Mn, Se, and Pb) could be involved in the pathogenesis of MBTs. However, the most noticeable change found was the elevated U content, indicating its considerable role as a major cerebral discriminator of the presence/absence of MBTs. The U/Se ratio could be considered as an appropriate blood marker in diagnostic MBT evaluation. The reported results could contribute to better understanding of the poorly understood pathogenesis of MBTs. Furthermore, the reported results could highlight a molecular basis for the pathophysiological changes caused by the hazardous effects of trace metals on brain homeostasis.

## Introduction

The importance of trace metals in the neurosciences has become more compelling during the first decade of the 21^st^ century^1^, as there is considerable interest in finding the relationships between trace metals and various neurological diseases, including central nervous system (CNS) malignancies. Metals show their harmful effects at the first stage of cancerogenesis (initiation)^2^. Initiation can affect normal biochemical reactions, leading to alterations in metal profiles. Considering that trace metals play a significant role in neoplastic processes, the metal contents in neoplastic samples can differ from the composition in control ones^3^. Homeostasis of metals within tight physiological limits is maintained through the mechanisms of uptake, distribution, accumulation, and secretion. Each breakdown has deleterious effects on metal-regulated metabolic pathways and can be a crucial step in the pathogenesis of various diseases. The most important pathway in metal-induced cancerogenesis is the ability of a metal to modulate gene expression by interfering with signal transduction pathways, leading to the deregulation of cell proliferation, activation of numerous transcription factors, disruption of cell cycle course, and apoptosis^4^.

Metal homeostasis is critical for the proper functioning of the brain^5^. The brain is a target organ for toxic environmental pollutants^6^. The balance of metals within the brain is regulated through the blood-brain barrier (BBB) and blood-cerebrospinal fluid (CSF) barrier. In physiological conditions, these barriers regulate CNS homeostasis and protect the CNS against hazardous chemicals. Damage to the endothelial structure of the BBB is a major cause of metal leakage into the surrounding brain parenchyma^7^. Based on metals’ abilities to pass through the BBB, it was hypothesized that prolonged exposure to metals could increase the risk of brain cancers, although no association between metal exposure and brain cancer was found^8^.

Primary malignant CNS tumors comprise 1.4% of all human cancers^9^. However, they are among the most lethal forms of human cancers due to their aggressive clinical evolution. The reported mortality rate of primary CNS tumors is approximately 60% in the first five years and they are ranked sixth among all malignancies^9–11^. Brain cancers are most frequently diagnosed in men, particularly the elderly^10^. The most common types of malignant brain tumors (MBTs) are gliomas, comprising approximately 80% of all primary brain neoplasms^12–14^. Glioblastomas are the most frequent and the most aggressive intraparenchymal MBTs in the adult population^15^.

Although metal analysis of liquid clinical samples cannot determine the role of metals in the pathogenesis of diseases, measurement of metals in serum, CSF, and other liquid samples can provide information related to metal deregulation in various diseases^16^. Analysis of trace metals in CNS tissues is the exact means to highlight the role of metals in the pathogenesis of neurological diseases. However, collecting brain tissues is difficult and feasible only after autopsy or surgery, considering that brain biopsy procedures are performed for histopathological examination of tumors in the diagnosis of cancer^5^. This could explain the insufficient number of studies in which metallomics has been utilized at the level of brain tissues.

The main objectives of this research were to assess whether trace metals could contribute to the pathogenesis of MBTs, and to examine if any of the selected metal ratios could be considered as a potential blood marker in the diagnostic evaluation of MBTs. In addition to its clinical relevance, the role of metal contaminants in the environment is also briefly discussed. In order to investigate metal profiles in the most representative way, various types of clinical samples were analyzed.

## Results

**Figure 1:**
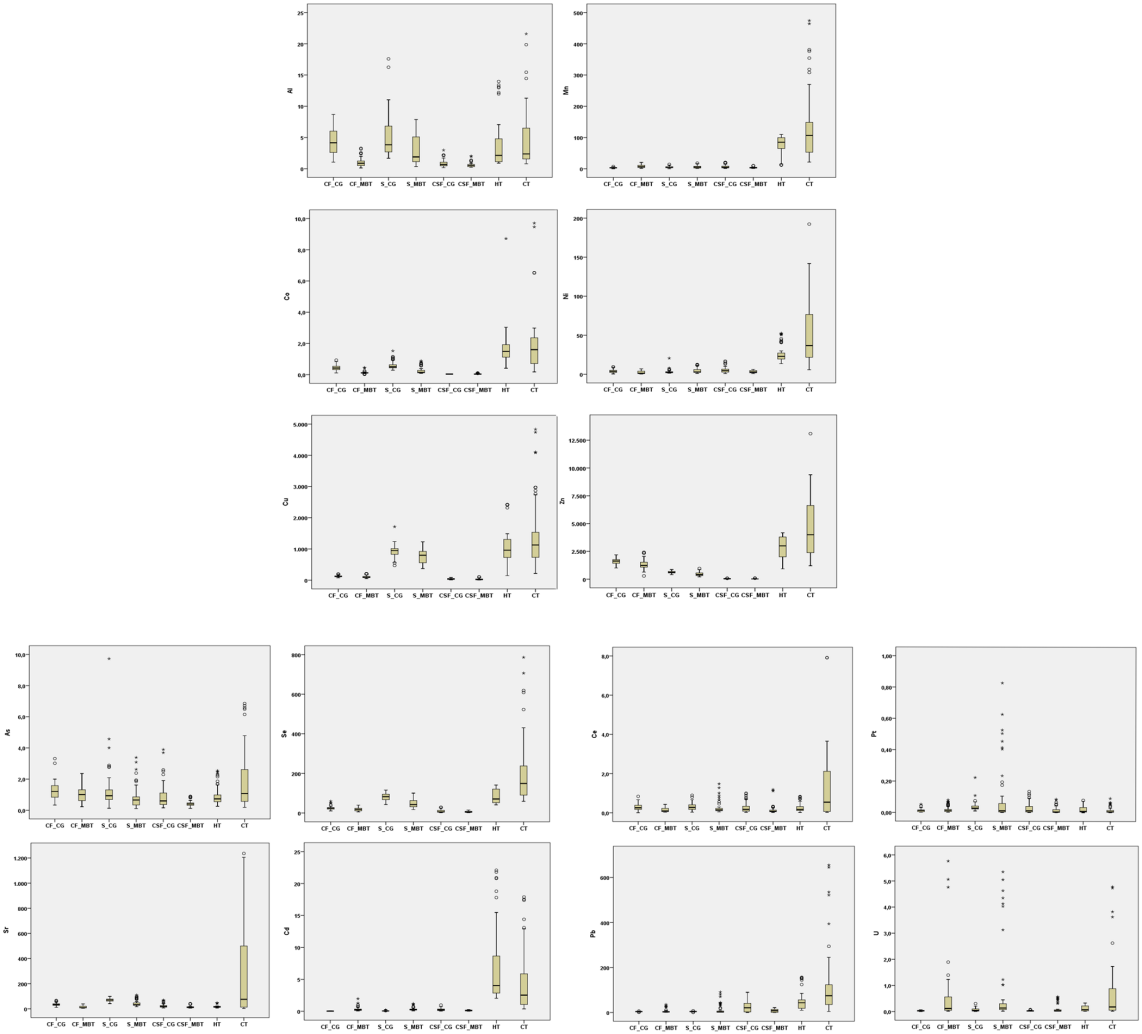
Box-plots for analyzed trace metals in the serum (S), cell fraction (CF), cerebrospinal fluid (CSF), healthy tissue (HT) and cancerous tissue (CT) samples of patients with malignant brain tumors (MBTs) in comparison to control group (CG).

The contents of metals in the analyzed samples are presented as mean ± standard deviation (st. dev.) and median values (Table 1). According to sex and age, the Chi-squared test showed no statistically significant difference between analyzed groups (p > 0.05). Therefore, the serum and cell fraction (CF) samples from MBT patients were compared to samples from blood donors, while CSF samples from MBT patients were compared to CSF samples from the hydrocephalus patients. The carcinogenic tissues (CTs) of patients were compared to healthy tissues (HTs) of the same patients. In this way, the most reliable comparison between groups was made. The box plots are given in Fig. 1. According to the Kolmogorov Smirnov’s test, the results for the metal contents showed skewed distributions, so differences between two groups were examined by the Mann-Whitney U-test (Table 1).

**Table 1:** The content of metals in serum, cell fraction (CF), cerebrospinal fluid (CSF), healthy tissue (HT) and cancerous tissue (CT) samples (ng/g).

		Serum		CF		CSF		Tissues	
		Control	Tumor	Control	Tumor	Control	Tumor	HT	CT
Al	mean	6.66	2.84	4.40	1.01	0.89	0.60	30.78	40.49
	st.dev.	2.84	2.36	2.47	0.73	0.68	0.41	40.10	50.15
	median	6.60	1.78	3.82	0.77	0.70	0.45	20.02	20.12
	*p-value*	0.069		0.097		0.123		0.087	
Mn	mean	4.71	10.77	3.06	7.55	3.47	6.05	77.83	132.64
	st.dev.	2.85	5.47	1.90	5.32	2.07	5.37	29.03	113.01
	median	3.93	6.55	2.79	6.29	3.10	3.97	80.66	105.83
	*p-value*	**0.039**		**0.044**		0.095		**0.019**	
Co	mean	0.47	0.25	0.43	0.14	0.04	0.04	1.63	2.18
	st.dev.	0.21	0.25	0.17	0.10	0.01	0.02	0.71	1.91
	median	0.47	0.12	0.43	0.12	0.03	0.03	1.58	1.36
	*p-value*	0.121		0.088		0.444		0.215	
Ni	mean	4.64	4.24	4.04	2.58	5.14	3.19	26.67	55.64
	st.dev.	2.30	3.16	2.45	2.09	3.81	1.68	11.41	47.75
	median	4.18	2.90	3.71	1.37	4.15	2.62	22.28	32.04
	*p-value*	0.321		0.302		0.114		0.065	
Cu	mean	949	788	125	109	39.99	30.90	1069	1399
	st.dev.	216	256	29	43	21.91	25.93	645	1192
	median	953	799	121	94	38.31	22.58	1063	1094
	*p-value*	0.089		0.796		0.111		0.099	
Zn	mean	619	461	1625	1318	39.16	27.80	2821	4699
	st.dev.	96	219	283	298	18.73	21.82	999	2902
	median	620	406	1625	1218	35.63	19.83	3014	3859
	*p-value*	**0.041**		**0.046**		**0.039**		**0.021**	
As	mean	1.54	0.86	1.26	1.03	0.86	0.43	0.92	1.85
	st.dev.	0.58	0.84	0.56	0.52	0.78	0.19	0.65	1.85
	median	1.51	0.65	1.17	0.91	0.49	0.41	0.70	0.84
	*p-value*	0.051		0.077		0.066		0.063	
Se	mean	82.43	46.33	24.94	12.96	8.99	6.16	83.71	200.23
	st.dev.	19.30	24.21	8.82	8.06	7.24	3.98	36.61	180.02
	median	82.65	37.67	24.67	13.60	5.78	4.99	66.60	146.48
	*p-value*	**0.024**		**0.033**		0.102		**0.003**	
Sr	mean	70.62	41.81	34.36	14.57	21.32	14.62	17.32	381.89
	st.dev.	11.73	25.41	11.86	11.41	11.48	9.63	10.81	252.87
	median	71.53	32.08	32.33	9.22	19.59	11.98	14.32	41.03
	*p-value*	**0.018**		**0.009**		**0.035**		**0.001**	
Cd	mean	0.02	0.31	0.01	0.31	0.26	0.12	6.71	4.63
	st.dev.	0.01	0.27	0.003	0.31	0.22	0.07	6.02	4.77
	median	0.02	0.25	0.01	0.18	0.18	0.10	3.65	2.75
	*p-value*	**0.000**		**0.005**		**0.012**		0.088	
Ce	mean	0.30	0.25	0.28	0.14	0.25	0.25	0.26	1.83
	st.dev.	0.20	0.36	0.16	0.12	0.25	0.16	0.24	1.33
	median	0.25	0.11	0.25	0.09	0.17	0.07	0.16	0.44
	*p-value*	**0.024**		**0.008**		**0.011**		**0.044**	
Pt	mean	0.03	0.21	0.01	0.02	0.03	0.02	0.02	0.03
	st.dev.	0.02	0.09	0.01	0.02	0.03	0.01	0.02	0.02
	median	0.02	0.04	0.01	0.01	0.01	0.00	0.01	0.01
	*p-value*	**0.002**		0.253		0.321		0.353	
Pb	mean	4.34	11.79	3.99	8.52	22.85	8.35	49.39	155.29
	st.dev.	1.07	4.44	0.89	5.60	20.67	7.87	36.11	120.45
	median	4.35	4.27	1.23	4.08	13.67	8.76	24.31	88.16
	*p-value*	**0.020**		**0.025**		**0.032**		**0.000**	
U	mean	0.06	1.52	0.03	1.27	0.02	0.16	0.11	1.13
	st.dev.	0.06	0.68	0.02	0.55	0.02	0.09	0.10	0.63
	median	0.05	0.12	0.04	0.14	0.01	0.03	0.06	0.21
	*p-value*	**0.001**		**0.000**		**0.000**		**0.000**	

The differences between groups were examined by Mann-Whitney U-test (at significance level 0.05). Statistically significant values are in bold.

**Figure 2:**
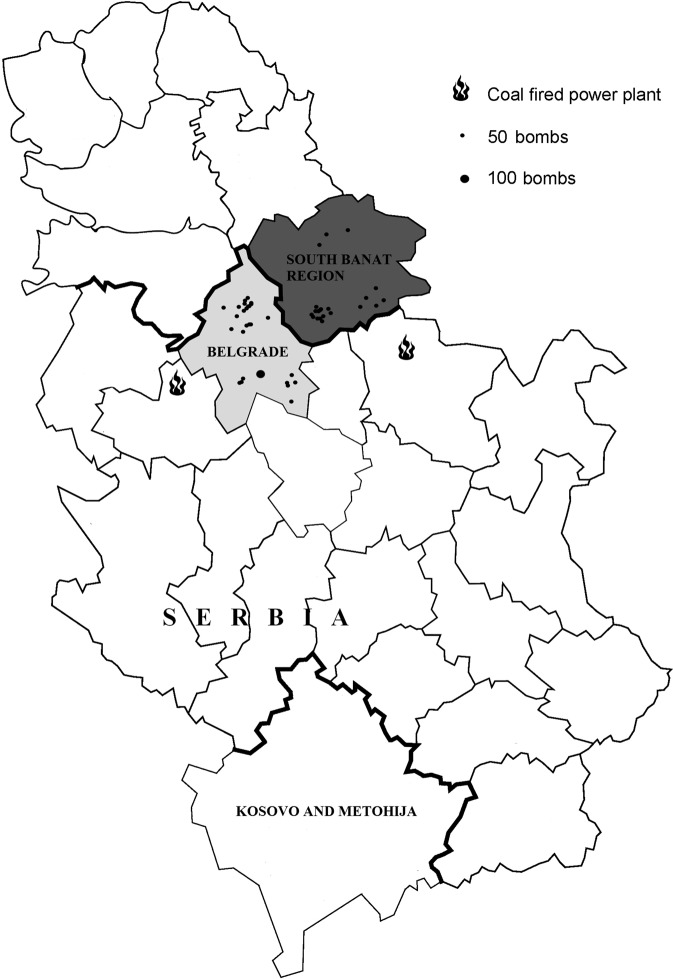
Map of Serbia. The shaded fields indicate the place of residence of the individuals as well as the potential causes of U exposure. The image was modified according to Stojsaljević *et al*.^34^ using Adobe Photoshop CS5 (Adobe Systems Incorporated, San Jose, CA, USA).

The most abundant metal in all samples was zinc (Zn), while the least abundant metal was platinum (Pt). Between the examined groups, there was no significant difference for aluminum (Al), cobalt (Co), nickel (Ni), copper (Cu), and arsenic (As) in any of the analyzed sample types (p > 0.05). Compared to control groups (CGs), MBT patients had significantly higher manganese (Mn) contents in serum (10.77 ± 5.47 vs. 4.71 ± 2.85 ng/g), CF (7.55 ± 5.32 vs. 3.06 ± 1.90 ng/g) and CT samples (132 ± 113 vs. 78 ± 29 ng/g), as well as higher lead (Pb) content in serum (11.79 ± 4.44 vs. 4.34 ± 1.07 ng/g), CF (8.52 ± 5.60 vs. 3.99 ± 0.89 ng/g) and CT samples (155 ± 120 vs. 49 ± 36 ng/g) (p < 0.05). Higher cadmium (Cd) content was found in the blood samples of MBT patients when compared to the CGs, i.e. in the serum (0.31 ± 0.27 vs. 0.02 ± 0.01 ng/g) and CF (0.31 ± 0.31 vs. 0.01 ± 0.003 ng/g). However, compared to the CGs, the Cd content was significantly lower in CSF (0.12 ± 0.07 vs. 0.26 ± 0.22 ng/g) and CT samples (6.71 ± 6.02 ng/g vs. 4.63 ± 4.77 ng/g) of MBT patients. In contrast, the contents of Zn, selenium (Se), strontium (Sr) and cerium (Ce) were significantly lower in the serum, CF and CSF samples (with the exception of Se and Ce in CSFs), but were significantly higher in the CT samples of MBT patients (p < 0.05). According to the median values, and in comparison with the CGs, the contents of Zn and Se were approximately 1.5-fold and 2.0-fold lower in both blood sample types and CSF samples of MBT patients, as well as 1.3-fold and 2.2-fold higher in the CT samples, respectively. The content of Sr was 2.2-, 3.5-, and 1.6-fold lower in the serum, CF and CSF samples of MBT patients in comparison to the CGs, respectively, as well as 2.9-fold higher in TC samples. The most significant metal that distinguished serum, CF, CSF and TC samples of MBT patients from the appropriate CGs was uranium (U). In general, the U content was up to 3.5-fold higher in all analyzed samples of MBT patients (p < 0.05) (Table 1 and Fig. 1) than in controls. According to Chi-squared test, the U contents in analyzed samples from patients in the different examined regions of Serbia did not differ significantly (Fig. 2).

The ratios between the metals that showed statistically significant differences, as well as the Cu/Zn ratio, were further investigated in order to find potential candidates that could distinguish each sample group, as well as to find an appropriate diagnostic marker for MBT patients. The results are presented in Table 2. The Cu/Zn ratio was unable to discriminate any of the sample types analyzed. The Zn/Mn and Pb/Mn ratios were significantly lower in the sera and CFs of MBT patients. The opposite results were obtained for Zn/Se and Pb/Se ratios (these were numerically higher in the sera and CFs of MBT patients), but there was no significance difference between groups (p > 0.05). For MBT patients, higher ratios of U/Mn and U/Se were able to discriminate the serum, CF, CSF and TC samples of MBT patients from those of the CGs, especially the higher ratio of U/Se in serum (P5-P95: 0.42–21.25 vs. 0.10–2.97 pg/kg) and CF (P5-P95: 0.70–30.35 vs. 0.19–3.73 pg/kg; p < 0.05), respectively (Table 2).

**Table 2:** The geometric mean (GM), percentiles (P), and the lower limit (LL) and upper limit (UL) of 95% confidence intervals for the ratios of metal contents (ng/g) in serum, cell fraction (CF), cerebrospinal fluid (CSF), healthy tissue (HT) and cancerous tissue (CT) samples.

		Serum		CF		CSF		Brain tissues	
		Control	Tumor	Control	Tumor	Control	Tumor	Control	CT
Cu/Zn	GM	1.51	1.79	0.08	0.08	0.97	1.04	0.32	0.26
	P5	0.94	1.03	0.05	0.04	0.49	0.38	0.16	0.10
	P25	1.27	1.35	0.06	0.06	0.70	0.90	0.31	0.14
	P50	1.50	1.67	0.07	0.07	0.85	1.16	0.37	0.21
	P75	1.78	2.27	0.09	0.10	1.33	1.32	0.45	0.49
	P95	2.65	3.01	0.12	0.51	2.34	2.03	0.58	0.78
	LL-UL	1.40–1.74	1.54–2.28	0.07–0.09	0.05–0.15	0.85–1.36	0.90–1.43	0.26–0.49	0.22–0.47
	*p-value*	0.222		0.339		0.247		0.169	
Zn/Mn	GM	149	91	573	195	6.9	7.6	31	36
	P5	62	36	241	47	2.2	3.7	17	13
	P25	90	64	397	116	3.8	5.2	25	20
	P50	140	98	544	224	6.7	7.5	38	39
	P75	216	132	720	304	9.0	10.9	40	48
	P95	461	194	2465	823	27.2	16.8	44	118
	LL-UL	134–222	78–136	483–903	160–329	6.2–13.4	6.1–12	24–41	30–61
	*p-value*	**0.033**		**0.001**		0.212		0.189	
Zn/Se	GM	7.6	10.2	68	74	5.2	4.5	34	27
	P5	4.9	4.7	39	23	1.4	2.3	16	8
	P25	5.9	6.7	55	55	2.2	3.1	31	17
	P50	7.4	9.8	65	72	7.2	4.2	33	28
	P75	10.4	15.8	91	101	9.1	7.4	49	40
	P95	12.3	21.3	138	201	12.7	9.3	60	72
	LL-UL	7.1–8.9	8.3–15.1	62–82	62–102	5–8.5	3.8–6.6	26–49	24–41
	*p-value*	0.144		0.332		0.412		0.214	
Pb/Mn	GM	1.00	0.49	1.37	0.48	2.00	1.18	0.53	0.61
	P5	0.29	0.15	0.22	0.06	0.07	0.13	0.18	0.08
	P25	0.55	0.29	0.98	0.21	0.31	0.20	0.28	0.25
	P50	1.13	0.57	1.52	0.37	4.70	2.50	0.50	1.01
	P75	1.63	0.68	1.96	1.52	8.66	3.86	0.86	1.49
	P95	2.98	2.26	6.78	2.88	18.99	5.82	1.82	2.28
	LL-UL	0.94–1.51	0.37–0.99	1.21–2.43	0.44–1.28	3.56–9.57	1.39–3.67	0.28–1.17	0.65–1.42
	*p-value*	**0.048**		**0.025**		0.058		0.236	
Pb/Se	GM	0.05	0.05	0.12	0.18	1.40	0.66	0.47	0.41
	P5	0.02	0.01	0.05	0.04	0.09	0.09	0.13	0.04
	P25	0.04	0.03	0.10	0.09	0.31	0.22	0.24	0.14
	P50	0.05	0.04	0.12	0.18	2.28	1.10	0.42	0.58
	P75	0.07	0.06	0.15	0.41	3.81	1.59	0.86	1.37
	P95	0.09	0.33	0.24	0.88	13.4	3.28	2.45	3.38
	LL-UL	0.05–0.06	≤0.23	0.11–0.15	0.15–0.39	1.6–5.9	0.7–1.67	0.12–1.46	0.48–1.41
	*p-value*	0.269		0.141		0.084		0.222	
U/Mn*	GM	9.36	18.41	9.35	16.84	4.32	9.30	1.05	1.73
	P5	0.71	4.51	0.93	0.74	1.54	2.15	0.28	0.26
	P25	5.74	6.22	5.69	3.75	3.33	3.86	0.36	0.50
	P50	10.88	16.68	10.19	17.14	4.56	6.06	0.69	1.49
	P75	14.35	29.52	20.22	86.31	5.69	15.82	3.23	4.65
	P95	119	197	62.62	296	11.30	169	6.72	30.1
	LL-UL	6.6–29	12.4–81	8.8–21.8	23.6–101	3.9–6.3	≤98.2	0.28–4.1	1.85–10.2
	*p-value*	**0.032**		**0.030**		**0.025**		0.128	
U/Se*	GM	0.51	2.42	1.09	6.52	3.01	5.55	0.93	1.16
	P5	0.10	0.42	0.19	0.71	1.12	1.01	0.26	0.18
	P25	0.32	1.03	0.77	1.70	2.09	1.72	0.33	0.38
	P50	0.55	1.62	1.21	5.43	2.72	4.46	0.61	0.94
	P75	0.78	7.76	1.93	30.30	3.44	9.81	3.38	4.75
	P95	2.97	21.25	3.73	56.43	10.55	98.42	5.11	12.42
	LL-UL	0.48–1.03	1.0–12.1	1.08–1.82	6.8–24	2.3–5.8	≤41.4	0.35–3.30	1.42–5
	*p-value*	**0.010**		**0.001**		**0.045**		0.658	

The differences between groups were examined by Mann-Whitney U-test (at significance level 0.05). Statistically significant values are in bold.

^*^Results presented in pg/kg.

## Discussion

### Comparative analysis

According to the results obtained, MBT patients had significantly altered metal content in serum, CF, CSF, and CT samples. Since relatively few papers on this topic have been published to the best of our knowledge, the possibility of providing adequate comparative analysis is limited. Arslan *et al*.^12^ reported higher contents of Cd, Mn, Pb and Zn, lower Cu content, and unchanged Co content in the serum of patients with malignant gliomas compared to that of healthy subjects. These data are in agreement with ours, with the exception of Zn. El-Yazigl *et al*.^17^ showed that the concentration ratio of malignant/control patients was 2.11 for Pb in CSF samples, and that there were no differences for Al, Cu, and Se ratios. These data agree with the results of our study. Andrasi *et al*.^18^ investigated several metals at the tissue level of glioblastoma patients and they reported no significant difference for tissue Cu between malignant and control samples, which is in agreement with the findings of the current study. Furthermore, the higher Sr content in glioblastoma brain tissues reported by Andrasi *et al*.^18^ is in agreement with the current study. Schrauzer^19^ reported that Co, Zn, and Se contents were higher in CT samples of MBT patients than in HTs. Our results support their findings. Civit *et al*.^2^ studied a high-grade oligodendroglioma tissue. Although only one sample was analyzed, there is agreement between their study and our current study regarding the contents of Cu, Co, As, Se, Mn, and Al and regarding the higher Zn content in CT. Recently, higher Zn content in samples of glioblastoma multiforme than in HT was reported by Wandzilak *et al*.^20^. Lankosz *et al*.^3^ showed the elemental profile of glial tissues could be applied to discriminate some types of brain tumors. Although relatively few studies have dealt with metal contents in MBTs, clear similarities between our current results and those reported previously for essential (Cu, Zn, Mn, Se, and Co) and toxic metals (Al, Sr, and As) was found.

### Essential metals in the brain

Brain metal homeostasis is achieved through specific transporters within the BBB, choroid plexus, hippocampus, and other regions, which can adequately deliver essential trace metals to the brain and remove excess metals from it. However, an excess of essential metals, like low levels of toxic metals, could induce neurological impairments^5^.

Copper is essential for normal brain function. The free Cu ion is transported through the BBB into the brain parenchyma, where it is utilized and released into the CSF^5^. Although an increased content of blood Cu has been associated with solid tumors, hematological malignancies^21,22^, and cancer progression (due to its key role in angiogenesis)^4^, no relationship between altered Cu level and brain cancers has been found. This is in agreement with the findings of the current study. The neuroprotective properties of Zn were demonstrated in the study by Jomova and Valko^4^. Due to its role in stabilization of myelin structure, Zn primarily accumulates in the white matter of the brain^5^. According to that study^5^, the reduced contents of Zn in liquid clinical samples and increased content in CTs could indicate the potential of malignant brain cells to withdraw essential Zn from the blood and/or CSF into the CT for the stimulation of cerebral tissue growth. Considering that the Cu/Zn ratio has been recommended as a suitable diagnostic blood marker for a great number of diseases, including various cancers^23^, we wanted to examine its potential role in the diagnostics of MBTs. The results of this research showed the Cu/Zn ratio has no role in discriminating our MBT samples from control samples, an important finding for excluding the Cu/Zn ratio from further evaluation.

It was previously reported that Mn mainly accumulated in the choroid plexus when the blood level of Mn was maintained at a constant^7^. Floriańczyk *et al*.^24^ mentioned the higher amount of Mn in the blood of MBT patients. However, the role of Mn in MBTs is not adequately understood. Our results showed that MBT patients had significantly elevated contents of Mn in their serum, CF, CSF and tissue samples, which implicates a role for the essential metal Mn in the stimulation of MBTs.

Although brain tissue is Se-deprived, Se deficiency can cause irreversible damage to the CNS^5^. In the current study, no Se deficiency was found in MBT patients. On the contrary, the investigated CTs contained significantly elevated Se levels. These results are very interesting, considering that our previous study highlighted the deficiency of Se in the thyroid tissue of the Serbian population^25^. Furthermore, in the current study, the Se concentrations in analyzed liquid clinical samples of MBT patients were significantly lower than in the CGs, which could signify the sequestration of Se into the malignant brain cells for the stimulation of cancer growth.

### Effects of toxic metals on brain homeostasis

This study demonstrated that the contents of Al, As, Ni, and Cd in CTs of MBT patients were not significantly different than the contents of these metals in the CG. Therefore, we suggest Al, As, Ni, and Cd could be eliminated from the point of view of MBT etiology. However, the high Pb content in serum, CFs, and CTs deserves further attention. It was reported that Pb, due to its negative effects on neurotransmitter pathways, could be harmful to brain tissue^5^. Investigations that considered Pb exposure and the risk of brain tumors are contradictory^26^. A recent investigation reported an increased risk of brain cancer with increasing Pb emissions from gasoline^8^. However, this information is of little importance for the Serbian population, since Pb was removed from gasoline over 30 years ago, according to international and national guidelines. In another study, no relationship was found between Pb and brain cancers^27^. According to the results obtained in our current study, the significantly elevated contents of Pb in the serum, CF and CT samples of MBT patients deserve further investigation.

Although Pb contents were able to discriminate MBT patients’ samples from the control samples, U acts as a major cerebral discriminator between samples from the ill and healthy groups. A possible explanation for the large U contents in the analyzed samples could be the several radioactive outbreaks that Europe experienced in recent decades, while Serbia (a part of the former Federal Republic of Yugoslavia) suffered directly from aerial bombardment in 1999. The U-based ammunition that was used during the air attacks on Serbia is the most recent source of U pollution released into the Serbian environment^28–31^. Uranium is primarily introduced into the body after inhalation and/or ingestion. Strongly oxidizing uranyl ion (UO_2_^2+^) in body fluids can form complexes, mainly with hydrogen-carbonate, which increases the solubility of U in blood^30,32^. Several studies reported concentrations of U in different types of blood samples. In a neighboring country, Croatia, the concentration of U in serum was 0.10–0.72 µg/L^32^, which is approximately 10-fold higher than the range previously reported in the literature (0.014–0.015 µg/L)^33^. Our previous study showed the healthy Serbian population had 0.03 to 0.20 µg/L of U in whole blood samples^34^. Dodorov *et al*.^35^ reported U concentrations of 0.14 to 0.80 µg/L in the blood of veterans exposed to depleted U in Gulf War I. Al-Hamzaei *et al*.^36^ analyzed whole blood samples from inhabitants from several locations that were centers of intensive military activities during Gulf wars. The authors reported a significantly higher mean concentration of U in the blood of cancer patients (2.62 ± 0.1 µg/L) compared to the CG (1.54 ± 0.1 µg/L). The present research showed the U concentrations in the serum (1.52 ± 0.68 ng/g) and CF (1.27 ± 0.55 ng/g) of MBT patients were similar to reported concentrations. The content of U in control and/or pathological CSF is unknown. As far as we can ascertain, this is the first study to show the content of U and the 13 other trace elements in CSF (Table 1).

Although the bones and kidneys are target organs for the manifestation of U toxicity, it was shown that U can cross the BBB and accumulate in the brain^6,37^. Imaging of U in different brain parts was studied by Zoriy *et al*.^38^. Accumulation of U is not uniform throughout the brain and it was demonstrated to be dose-dependent^37^. Al-Hamzaei *et al*.^36^ reported that the increased chemical activity of U could cause DNA mutations, and potentially, cancerogenesis. However, the issue of U toxicity remains unclear.

It is worth noting that all analyzed samples in the current study were collected in the period 2016–2019, and that the ICP-MS instrument used in this study cannot provide information on whether depleted U was detected in our samples, due to the inability of quadrupole (Q) to accurately separate ^235^U from ^238^U. However, the high U contents in sera, CF, CSF and CT samples from MBT patients and the contents occurring in healthy subjects should be further investigated in clinical studies.

For the first time, this study showed that MBT patients had altered profiles of some metals in serum, CF, CSF, and CT samples when compared to control samples. Moreover, the higher contents of Mn, Se, and Pb in MBT patients could indicate these metals are involved in the pathogenesis of MBTs. However, the most noticeable change found was the elevated U content, indicating its considerable role as a major cerebral discriminator of the presence/absence of MBTs. The U/Se ratio could be considered as an appropriate blood marker in diagnostic MBT evaluation. The results of this research could lead to a better understanding of the roles of essential and toxic trace metals in the inadequately understood pathogenesis of MBTs. Furthermore, the obtained results could indicate a molecular basis for the pathophysiological changes stemming from the hazardous effects of trace metals on brain homeostasis and dysfunction.

## Material and Methods

### Sample collection

The research included 61 patients with MBTs (female/male ratio = 25/36; mean age: 40 ± 4), 51 patients with hydrocephalus (female/male ratio = 24/27; mean age: 47 ± 5), and 61 healthy blood donors (female/male ratio = 30/31; mean age: 45 ± 6).

Intravenous blood from blood donors and patients with MBTs was collected in trace metal-free evacuated tubes (BD Vacutainer). The collected whole blood (5 mL) from each subject was left for approximately 30 min and the obtained serum was separated from the CF after centrifugation (3000 × g). The serum and CF samples were immediately frozen at −80 °C. CSF was collected by ventricular puncture during a surgical shunt procedure in patients with hydrocephalus. Brain tissues were collected during surgery. Preoperative diagnosis was carried out by applying imaging techniques, such as X-ray, computed tomography, and/or magnetic resonance imaging (MRI). A definitive diagnosis of MBT was confirmed by two neuropathologists after postoperative histopathological analysis of brain tissue. The standard technique for surgical excision of CT included the peritumoral margin of normal cerebral tissue. This tissue was collected and considered as HT after the exclusion of tumor by histopathological examination. HT samples we utilized as defined self-controls for each patient’s tumor tissue samples. All tumors were classified according to the criteria defined by the World Health Organization (WHO). The following types of MBT were included in the research: 54 glioblastoma multiforme grade IV, three medulloblastoma grade III, and four atypical teratoid rhabdoid tumors. All collected samples were stored at −80 °C until analysis.

In order to avoid confounding factors in the distribution of metals, smokers and patients who had other malignancies or liver or kidney failure were excluded from this research. Approval was obtained by the Ethics Committee of Clinical Centre of Serbia, Belgrade. All patients and blood donors voluntarily participated in this research, and written informed consent from all study participants was obtained.

All methods were conducted following relevant guidelines and regulations.

### Chemicals, instrumentation and analysis

High grade concentrated nitric acid and hydrogen peroxide were supplied by Merck (Darmstadt, Germany). Nitric acid was additionally purified using the Berghof-acid purification apparatus-BSB-939-IR. Ultrapure water (resistance of 18.2 MΩ) was obtained by Milli Q Plus System (Merck, Darmstadt, Germany). A certified multi-element standard solution containing 22 elements (10 mg/L) and an internal standard solution (100 mg/L ^7^Li, Sc; 20 mg/L Bi, Ga, In, Tb, Y) were supplied by VHG, Manchester, UK. Instrumental parameters for ICP-MS were optimized using iCAP Q tuning solution containing 1 µg/L of Ba, Bi, Ce, Co, In, Li and U in 2% nitric acid (Thermo Scientific, UK). Standard reference materials (SRMs) of whole blood (SERO210105, Level 1, and SERO210305, Level 3) (Seronorm, Sero AS, Norway), and bovine liver (1577c, NIST, US) were employed for analytical quality assurance. Samples were digested using a microwave digestion system (ETHOS 1, Milestone, Italy).

All elements were quantified by inductively coupled plasma-mass spectrometry, ICP-MS (iCAP Q_*c*_, Thermo Scientific, UK). Pure argon (99.999%), supplied by Messer (Pančevo, Serbia) was used for plasma formation and dispersion of diluted samples. The collision cell of ICP-MS was filled with high purity helium gas (99.999%), also supplied by Messer. All measurements on ICP-MS were performed in the optimized interference mode, which is based on kinetic energy discrimination. In order to compensate matrix-induced ion signal fluctuations and instrumental drift, a solution containing ^45^Sc at a concentration of 50 µg/L and ^71^Ga, ^89^Y, ^115^In, ^159^Tb, ^208^Bi at concentrations of 10 µg/L was equally distributed by a second channel of the peristaltic pump in blank, standard solutions, and samples. By applying six calibration solutions in a range from 1 to 500 µg/L, the linearity of the calibration curve for each element was greater than 0.999. Intra-day precision was estimated by calculating the relative standard deviation (RSD) at three different standard concentrations (5, 50 and 100 μg/L) in 6 replicates. Inter-day precision was estimated for the same calibration solutions (6 replicates over 6 consecutive days). Intra-day and inter-day RSDs were 2.5% and 3.7%, respectively. The limit of detection (LOD) and limit of quantification (LOQ) were determined based on the mean and standard deviation of a blank. Three and ten determinations were made with a blank solution (at no concentration in the appropriate matrix) to set the LOD and LOQ, respectively. The accuracy of the analytical method was controlled by SRMs. Based on the accuracy obtained with SRMs, the following isotopes were selected: ^27^Al, ^55^Mn, ^59^Co, ^60^Ni, ^65^Cu, ^66^Zn, ^75^As, ^82^Se, ^88^Sr, ^111^Cd, ^142^Ce, ^194^Pt, ^208^Pb, and ^238^U. The obtained recovery values for elements in SRMs were in the range of 91.2 to 108.9%.

### Microwave digestion of samples

Approximately 0.5 g of sample was transferred into a microwave vessel and the exact weight was measured. All samples were digested at 180 °C in a 4:1 (v/v) mixture of nitric acid (65%) and hydrogen peroxide (30%). Microwave digestion was performed according to the following mode of operation: 5 min warm-up to 180 °C and hold at 180 °C for an additional 20 min. After a cooling period, digested samples were transferred into 25 mL-volumetric flasks and diluted with ultrapure water. The SRMs were firstly reconstituted according to the manufacturers’ instructions and further prepared as clinical samples.

### Data analysis

Descriptive statistics, Box plots, Chi-squared test, Kolmogorov Smirnov’s test, and Mann Whitney U-test were performed using SPSS statistical software (IBM Statistics 20). In all applied statistical tests, the significance level was 0.05. According to the recommendations of International Federation of Clinical Chemistry and Laboratory Medicine (IFCC) and the International Union for Pure and Applied Chemistry (IUPAC), the ratio between metals was expressed as a percentile (P) in the range of the 5^th^–95^th^ percentiles, and it was calculated as the lower limit (LL) and upper limit (UL) of the 95% confidence interval (CI).
